# Emerin interacts with histone methyltransferases to regulate repressive chromatin at the nuclear periphery

**DOI:** 10.3389/fcell.2022.1007120

**Published:** 2022-10-06

**Authors:** Nicholas Marano, James M. Holaska

**Affiliations:** Department of Biomedical Sciences, Cooper Medical School of Rowan University, Camden, NJ, United States

**Keywords:** emerin, Emery-Dreifuss muscular dystrophy, myogenic differentiation, repressive chromatin, EZH2, G9a

## Abstract

X-Linked Emery-Dreifuss muscular dystrophy is caused by mutations in the gene encoding emerin. Emerin is an inner nuclear membrane protein important for repressive chromatin organization at the nuclear periphery. Myogenic differentiation is a tightly regulated process characterized by genomic reorganization leading to coordinated temporal expression of key transcription factors, including MyoD, Pax7, and Myf5. Emerin was shown to interact with repressive histone modification machinery, including HDAC3 and EZH2. Using emerin-null myogenic progenitor cells we established several EDMD-causing emerin mutant lines in the effort to understand how the functional interaction of emerin with HDAC3 regulates histone methyltransferase localization or function to organize repressive chromatin at the nuclear periphery. We found that, in addition to its interaction with HDAC3, emerin interacts with the histone methyltransferases EZH2 and G9a in myogenic progenitor cells. Further, we show enhanced binding of emerin HDAC3-binding mutants S54F and Q133H to EZH2 and G9a. Treatment with small molecule inhibitors of EZH2 and G9a reduced H3K9me2 or H3K27me3 throughout differentiation. EZH2 and G9a inhibitors impaired cell cycle withdrawal, differentiation commitment, and myotube formation in wildtype progenitors, while they had no effect on emerin-null progenitors. Interestingly, these inhibitors exacerbated the impaired differentiation of emerin S54F and Q133H mutant progenitors. Collectively, these results suggest the functional interaction between emerin and HDAC3, EZH2, and G9a are important for myogenic differentiation.

## Introduction

X-linked Emery-Dreifuss muscular dystrophy (X-EDMD; EDMD1) is caused by mutations in the gene encoding emerin (Bione et al., 1994). Emerin is an integral inner nuclear membrane (INM) protein with putative roles in repressive chromatin organization at the nuclear periphery (Demmerle et al., 2012, 2013; Ranade et al., 2019). Patients with EDMD1 experience skeletal muscle wasting and dilated cardiomyopathy (Heller et al., 2020). Skeletal muscle wasting may be attributed to the inability of the resident muscle stem cell pool to differentiate and regenerate damaged muscle (Koch and Holaska, 2014). Upon muscle injury or mechanical stimulation, these muscle stem cells (or myogenic progenitor cells) initiate the myogenic differentiation program, which is a tightly regulated process driven by the coordinated temporal expression of key transcription factors. Genomic reorganization is required for this transcriptional reprogramming during commitment to differentiation and formation of myotubes (Bharathy et al., 2013).

Purification of emerin-containing complexes from HeLa cells revealed interactions with chromatin associated proteins, such as barrier-to-autointegration factor (BAF) and histone deacetylases (HDAC) 1 and 3 (Holaska and Wilson, 2007). Emerin binds histone deacetylase 3 (HDAC3) in C2C12 myoblasts and in HeLa cells (Demmerle et al., 2012). HDAC3 is a class I lysine deacetylase and the catalytic subunit of the Nuclear CoRepressor complex (NCoR) (Moser et al., 2014) responsible for deacetylation of H4K5 (Bhaskara et al., 2010). *In vitro* studies showed that emerin binds directly to HDAC3 and activates its catalytic activity (Demmerle et al., 2012). Emerin-null myogenic progenitors showed a significant increase in transcriptionally active H4K5ac modification, which is the target of HDAC3 (Collins et al., 2017). Similar results were seen in emerin-downregulated HeLa cells (Demmerle et al., 2012). Differentiating emerin-null myogenic progenitors also had increased H4K5ac in promoters of key differentiation genes. Emerin-null progenitors also failed to coordinate the spatiotemporal localization of differentiation gene loci to the nuclear envelope, resulting in maintenance of their expression (Demmerle et al., 2013). Treatment with an HDAC3 activator rescued *Myf5* localization and myogenic differentiation (Demmerle et al., 2013), demonstrating the functional interaction between emerin and HDAC3 is important for differentiation. Treatment with histone acetyltransferase inhibitors specifically targeting H4K5 acetylation also rescued emerin-null differentiation (Bossone et al., 2020). We hypothesize that emerin is important for maintaining H4K5ac homeostasis during myogenic differentiation to ensure proper transcriptional reprogramming. It is possible emerin also regulates the coordinated deposition of H3K9me2/3 and H3K27me3 during differentiation.

Interestingly, EDMD1-causing emerin mutants (S54F, Δ95-99, Q133H, P183H) all failed to bind HDAC3 (Demmerle et al., 2012). Myogenic progenitors expressing the EDMD1 mutants in an emerin-null background exhibited impaired differentiation (Iyer and Holaska, 2020) suggesting the interaction of emerin with HDAC3 is vital for myogenic differentiation. Similar molecular pathways were disrupted in each of the EDMD1 mutants and emerin-null cells (Iyer et al., 2017; Iyer and Holaska, 2020), suggesting similar mechanisms underly impaired differentiation in EDMD1. These observations suggest the emerin-HDAC3 interaction may be important for the impaired muscle regeneration seen in EDMD1 patients.

Msh homeobox 1 (Msx1) has been shown to colocalize with polycomb repressive complex 2 (PRC2) and H3K27me3 at the nuclear periphery in C2C12 cells and primary myoblasts (Wang et al., 2011). Emerin is responsible for the recruitment of Msx1 and EZH2 to the nuclear envelope (Ma et al., 2019). EZH2, the catalytic component of PRC2, mediates the trimethylation of H3K27 (Tan et al., 2014)—a repressive histone modification reported to be enriched in lamina-associated domains (van Steensel and Belmont, 2017). ChIP-Seq experiments show H3K27me3 enrichment and repression of transcriptional regulators Myf5 and MyoD is dependent upon Msx1 (Wang et al., 2011). Localization of Msx1 and EZH2 at the nuclear envelope is emerin-dependent, as emerin-knockdown in C2C12 myoblasts caused both Msx1 and EZH2 localization to be lost at the inner nuclear membrane (Wang and Abate-Shen, 2012b; Ma et al., 2019).

Msx1 can also recruit euchromatic histone lysine methyltransferase 2 (EHMT2 or G9a) in C2C12 cells (Wang and Abate-Shen, 2012a). H3K9me2 enrichment at regulatory regions of key myogenic transcription factors is dependent on Msx1 (Wang and Abate-Shen, 2012a). G9a functions primarily as a heterodimer with lysine methyltransferase G9a-like protein (GLP) (Tachibana et al., 2005). Although G9a and GLP are structurally similar, they do serve distinct roles in myogenesis. Exogenous expression of G9a was shown to impair muscle differentiation in C2C12 cells (Ling et al., 2012), whereas knockdown of G9a favors muscle differentiation (Battisti et al., 2016). G9a was also shown to interact with the PRC2 complex (e.g., EZH2) and G9a knockdown led to decreased EZH2 and H3K27me3 at PRC2 target genes (Mozzetta et al., 2014). G9a and PRC2 may cooperate to organize repressive chromatin at the nuclear envelope and regulate myogenic differentiation, as genomic studies have suggested colocalization of PRC2 and H3K9 methyltransferases (Wang et al., 2008).

Collectively, these data support a model whereby emerin modulates repressive chromatin reorganization at the nuclear envelope during transcriptional reprogramming. How this occurs remains unknown. Thus, we set out to better understand the functional interaction between emerin, HDAC3, and EZH2 and G9a, and its role in myogenic differentiation. Here we show emerin interacts with both G9a and EZH2 in myogenic progenitors. We further demonstrate that emerin HDAC3-binding mutants show enhanced binding to G9a and EZH2, resulting in more H3K27me3 and H3K9me2 at the nuclear envelope and impaired differentiation. Using EZH2 and G9a inhibitors, we show these functional interactions are important for myogenic differentiation.

## Methods

### Cell culture

Myogenic progenitors from H2K wildtype and EMD^−/y^ mice were obtained from Tatiana Cohen and Terence Partridge (Children’s National Medical Center, Washington DC, United States). Proliferating myogenic progenitors were counted in a Countess^®^ II FL Automated Cell Counter (ThermoFisher Scientific), plated at 650 cells/cm^2^, and incubated in proliferative media consisting of high-glucose DMEM (ThermoFisher Scientific) supplemented with 2% chick embryo extract (Accurate Chemical; #MDL-004-E), 20% heat-inactivated FBS (ThermoFisher Scientific; #MDL-004-E), 1% penicillin-streptomycin (ThermoFisher Scientific), 2% L-glutamine (ThermoFisher Scientific) and 20 units/mL γ-interferon (MilliporeSigma) at 33°C and 10% CO_2_, as previously described (Iyer and Holaska, 2020). Emerin-null progenitor lines and emerin-null cell lines expressing wildtype emerin (+EMD), emerin EDMD1 mutants Q133H or S54F emerin, or vector alone control cell lines were previously established and grown in the presence of 6 μg/ml puromycin, 10 μg/ml puromycin, and 15 μg/ml puromycin, respectively (Iyer and Holaska, 2020). For differentiation experiments, cells were plated at 25,000 cells/cm^2^ in proliferative media at 33°C and 10% CO_2_. Cells were washed once with PBS and incubated with differentiation media consisting of high-glucose DMEM, 2% horse serum, and 1% penicillin/streptomycin at 37°C and 5% CO_2_.

### Co-immunoprecipitation and western blotting

Dynabeads™ protein G magnetic beads (ThermoFisher Scientific; #1007D) were washed once with washing buffer. Beads were then resuspended in antibody binding buffer and incubated with antibodies against emerin (Proteintech, #10351-1-AP), G9a (Abcam; #185050), or EZH2 (Active Motif #39239) overnight at 4°C with rotation. Wildtype and emerin-null progenitors expressing S54F or Q133H EDMD1 mutants were grown in 15 cm dishes coated with 0.01% gelatin in proliferative conditions, as described above. Cells were incubated with 0.05% Trypsin-EDTA (Gibco; #25300054) for 5 min at 37°C and 10% CO_2_ and collected by centrifugation (160 g, 5 min). Cell pellets were lysed on ice in modified NEHN buffer (20% glycerol, 500 mM NaCl, 20 mM HEPES, 1 mM PMSF, 1 mM EDTA, 2 mM DTT, 1% NP-40, aprotinin, leupeptin, and pepstatin A). Whole cell lysates were then sonicated on ice with 30 s on/off intervals for 10 min and placed on ice for 5 min. This was repeated three more times. Lysates were spun at 16,300 g at 4°C for 20 min. Insoluble components were pelleted and the soluble fraction was collected. Antibody-bound Dynabeads™ protein G magnetic beads were washed twice with washing buffer and incubated overnight with soluble fractions. Beads were separated from the soluble fraction with a magnet and resuspended in washing buffer for three 5-min washes with rotation. Bound complexes were eluted from beads per manufacturer instructions (ThermoFisher Scientific). Eluted fractions were incubated with 1:1 mixture of 2X NuPage™ LDS sample buffer at 75°C and 500 rpm for 10 min on a hot plate mixer (ThermoFisher Scientific; #NP0007). Samples were loaded and separated by SDS-PAGE at 150 V for 50 min. Gels were transferred to nitrocellulose membranes at 250 mA for 75 min on ice with stirring. Membranes were blocked with 5% non-fat milk (Giant Food Stores) in phosphate buffered saline (pH 7.4) containing 0.1% Tween-20 (PBST) for 1 hour at room temperature followed by incubation with antibodies against emerin (1:3,000; Leica Biosystems; #NCL-EMERIN), G9a (1:5,000; Proteintech; #66689-1-Ig) or EZH2 (1:5,000; Proteintech; #66476-1-Ig) diluted in 0.5% non-fat milk overnight at 4°C with mild shaking. Blots were washed with PBST at least 3 times for 5 min at room temperature and incubated with HRP-conjugated secondary antibodies against mouse IgG (ThermoFisher Scientific; #31432). Membranes were then washed with PBST at least 3 times for 5 min. Blots were incubated with SuperSignal™ West PicoPlus chemiluminescent substrate solution (Life Technologies; #34580) and visualized. Images were taken on a Bio-Rad ChemiDoc MP imaging system. Densitometry was performed using Bio-Rad Image Lab^®^ software. Ratios of bound to input were measured and plotted. Input and unbound sample lanes represent 2.5% of total. Bound lanes represent 50% of input.

### Immunofluorescence microscopy

Myogenic progenitors were plated on gelatin-coated coverslips or directly in gelatin-coated 96-well dishes for immunofluorescence microscopy. Proliferative cells were plated on gelatin-coated coverslips and grown for 2 days (30%–40% confluent) at 33°C, 10% CO_2_. Differentiation samples were plated at 25,000 cells/cm^2^ and incubated in proliferative media overnight at 33°C and 10% CO_2_. Coverslips and 96-well dishes were washed three times with phosphate buffered saline (PBS, pH 7.4), fixed with 3.7% formaldehyde in PBS for 15 min, washed once with PBS for 5 minutes at room temperature, permeabilized with PBS containing 0.2% Triton X-100 for 20 min at room temperature, followed by washing once with PBS for 5 minutes at room temperature. All washing and incubation steps are performed with moderate shaking. Coverslips or 96-well plates were blocked in PBS containing 3% BSA and 0.1% Triton X-100 for 2 h at room temperature, followed by incubation with antibodies against emerin (1:300; Leica Biosystems; #NCL-Emerin), H3K9me2 (1:250: Active Motif; #39239), G9a (1:500; Abcam; #185050), H3K27me3 (1:200; Millipore Sigma; #MP 07–449) or EZH2 (1:250; Active Motif; #39901) overnight at 4°C with shaking. Coverslips were washed three times in PBS at room temperature and incubated with Alexa Fluor™-conjugated secondary antibodies against rabbit or mouse IgG (Invitrogen) for 1 h at room temperature shielded from light. All primary and secondary antibodies were diluted in PBS containing 3% BSA and 0.1% Triton X-100. Coverslips were mounted on slides with ProLong™ Diamond Antifade Mountant with DAPI (Life Technologies; #P36971). Myogenic differentiation was done in 96-well plates because myotubes can more readily detach from coverslips. For immunofluorescence microscopy on differentiating cells, the cells were plated at 25,000/cm^2^ (differentiation density) and incubated overnight in proliferation media at 33°C and 10% CO_2_. Wells were washed with PBS and differentiation media was added to each well and the cells were incubated for 24–72 h at 37°C, 5% CO_2_. Blocking, fixing, washing, and incubating with primary and secondary antibodies was done as described for the coverslips. 96-well dishes were incubated with 0.2 μg/ml DAPI in PBS for 5 min and washed twice with PBS.

### Confocal microscopy

Slides of myogenic progenitors were prepared as described above and confocal images were taken using a Nikon Eclipse Ti2-E Inverted Research Microscope equipped for confocal, brightfield and DIC imaging in conjunction with a Nikon A1rSi Laser Point Scanning Confocal System. All images were taken with a 60X oil-immersion objective (N.A. = 1.4). Nikon Imaging Software (NIS) Elements was used to record images. Each field was subject to the same light conditions and exposure times. At least 30 nuclei were imaged for each trial. Files were analyzed in NIS Elements Viewer software and ImageJ. Localization was measured using 21.1 μm lines that were drawn across similar axes of nuclei using ImageJ software. Fluorescence was quantified along the superimposed line in each field and measurements were plotted on the same graph.

### Histone methyltransferase inhibition and myogenic differentiation assay

2.5 µM G9a inhibitor UNC0638 (BioVision; #1933) or 4.0 µM EZH2 inhibitor GSK126 (BioVision; #2282) were added to differentiation media upon differentiation induction (t = 0). At 24, 48, or 72 h after induction of differentiation, the cells were lysed in SDS-PAGE sample buffer, separated by SDS-PAGE, and western blotting was done using antibodies against H3K9me2 (1:2,000; Active Motif; #39239), H3K27me3 (1:1,500; Millipore Sigma; #MP 07–449), or γ-tubulin (1:10,000; ThermoFisher, Scientific). The levels of H3K9me2 and H3K27me3 were normalized to γ-tubulin expression and analyzed as described above. Validation of inhibitors was performed in all cell lines and two biological replicates were prepared for each treatment at each time point.

The ClickIt EdU reaction kit (ThermoFisher Scientific; #C10640) was used to assess cell cycle withdrawal. EdU was added to media 2 h prior to washing with PBS and fixing with 3.7% formaldehyde in PBS for 15 min. Cells were washed once with PBS, permeabilized with 0.2% Triton X-100 in PBS for 20 min at room temperature, and stained per manufacturer instructions. Nuclei were incubated with 0.2 μg/ml DAPI in PBS for 5 min at room temperature. Images of EdU (Alexa-647) and DAPI (405 nm) were taken using a 40X objective (N.A. = 0.65) on an Evos FL Auto 2 microscope. Differentiation was quantified by monitoring the expression of myosin heavy chain (MyHC) using MyHC antibodies (1:20; Santa Cruz Biotechnology; #SC-376157). A minimum of three biological replicates were prepared for each condition and at least three images were taken (random location) for each biological replicate in each field. At least 75 nuclei were imaged for each trial. Images were counted using the cell counter plugin on ImageJ and analyzed in Prism 9 software. Percentages of EdU-positive or MyHC-positive cells were compared to total nuclei (DAPI) for each replicate. Fusion index was determined by merging DAPI and MyHC channels and counting cells containing at least 3 nuclei sharing MyHC-stained cytoplasm.

## Results

### Emerin HDAC3-Binding mutants bind more G9a and EZH2

Our previous data suggests that emerin binding to HDAC3 results in more chromatin deacetylation at the nuclear envelope which could recruit HMTs (e.g., EZH2 and G9a) to methylation targets and more stably repress nuclear envelope-associated chromatin. Emerin was reported to interact with EZH2 *via* Msx1, so we tested whether emerin and the EDMD1 mutants could bind to EZH2. H3K9me2 has also been implicated in organizing repressive chromatin at the nuclear envelope, so we also tested if emerin could bind G9a.

**FIGURE 1:**
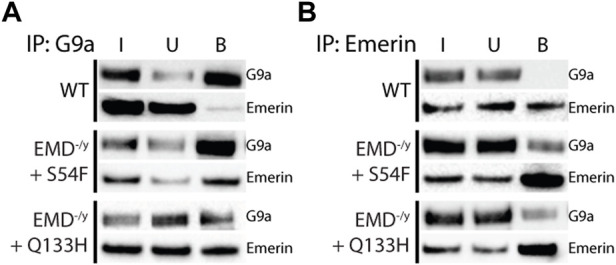
Emerin mutants that fail to bind HDAC3 bind more G9a. Immunoprecipitation of G9a or emerin was performed in WT or emerin-null progenitors expressing emerin mutants S54F or Q133H. **(A,B)** Protein G magnetic beads were incubated with anti-G9a antibody (A; *n* = 3 for all cell lines) or anti-emerin antibody (B; *n* = 3 for all cell lines), followed by incubation with whole-cell lysates from each cell line. Input (I), unbound (U), and bound (B) fractions were separated by SDS-PAGE, transferred to nitrocellulose, and incubated with antibodies against emerin and G9a.

**FIGURE 2:**
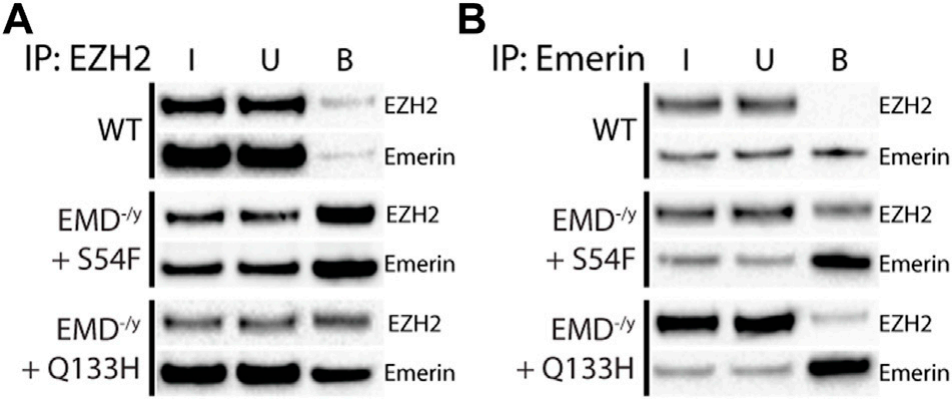
Emerin mutants that fail to bind HDAC3 bind more EZH2. Immunoprecipitation of EZH2 or emerin was performed in WT or emerin-null progenitors expressing emerin mutants S54F or Q133H. **(A,B)** Protein G magnetic beads were incubated with anti-EZH2 antibody (A; *n* = 3) or anti-emerin antibody (B; *n* = 3), followed by incubation with whole-cell lysates from each cell line. Input (I), unbound (U), and bound (B) fractions were separated by SDS-PAGE, transferred to nitrocellulose, and incubated with antibodies against emerin and EZH2.

Co-immunoprecipitation experiments with antibodies against emerin, EZH2, or G9a were done on wildtype myogenic progenitors (*n* = 3) or emerin-null progenitors expressing either emerin S54F (*n* = 3) or Q133H (*n* = 3) at wildtype levels. A small fraction of endogenous emerin bound G9a (Figure 1A; S1, 0.76% of input), whereas G9a bound a much larger fraction of S54F (80.9% of input) and Q133H (45.1% of input). Reciprocal experiments were done using antibodies against emerin for the immunoprecipitation and similar results were seen with 0.2% of G9a binding to wildtype emerin (*n* = 3) and 13.8% and 9.6% binding to S54F (*n* = 3) and Q133H (*n* = 3), respectively (Figure 1B; Supplementary Figure S1). We next analyzed emerin binding to EZH2 by co-IP in myogenic progenitors and S54F and Q133H emerin mutant myogenic progenitors. Immunoprecipitation with antibodies against EZH2 showed 0.7% emerin bound EZH2 in wildtype progenitors (Figure 2A; Supplementary Figure S2; *n* = 2), whereas 71.8% of S54F (*n* = 3) and 77.8% of Q133H (*n* = 2) bound EZH2. Similar results were seen in the reciprocal immunoprecipitation, with 2.4% EZH2 bound to wildtype emerin (*n* = 3), and 57.9% and 89.4% of EZH2 binding to S54F (*n* = 3) and Q133H (*n* = 2), respectively (Figure 2B; Supplementary Figure S2).

### More H3K9me2 and H3K27me3 localize to the nuclear envelope in S54F and Q133H myogenic progenitors

**FIGURE 3:**
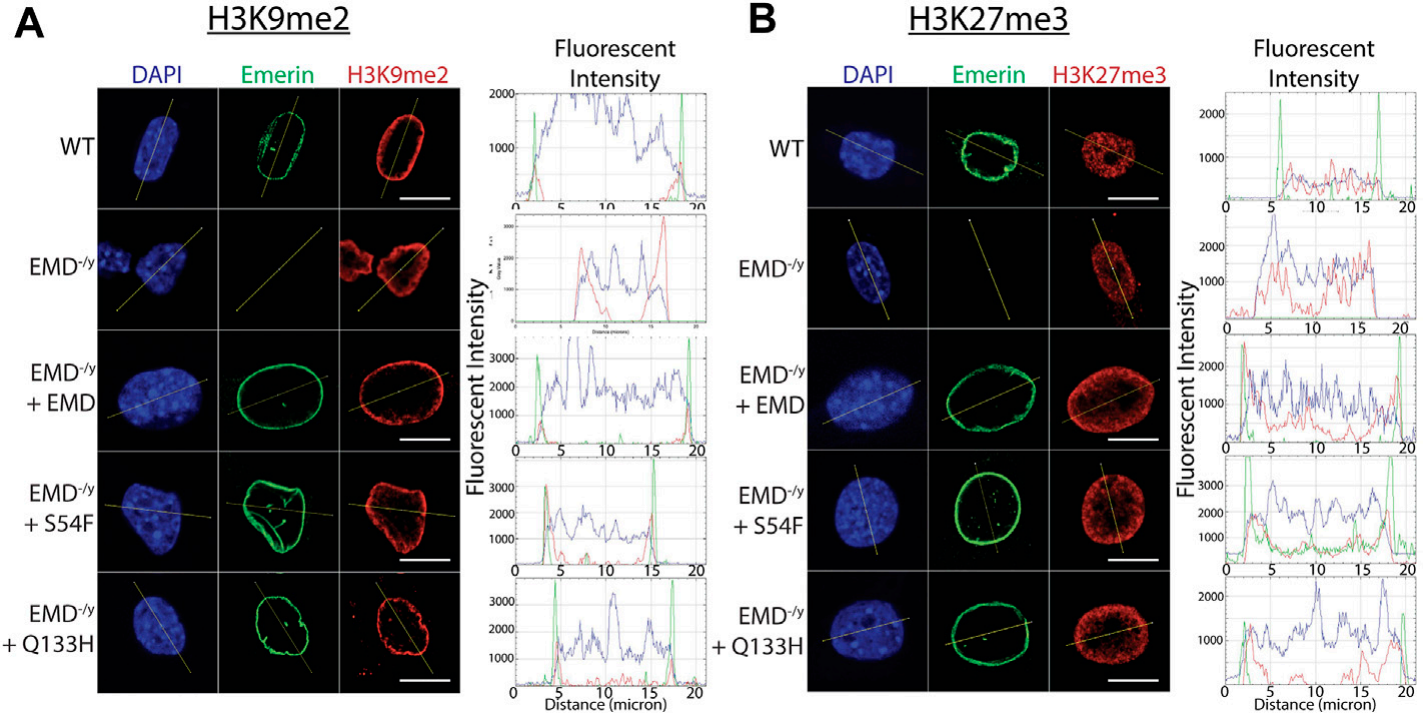
Confocal imaging shows enrichment of H3K9me2 and H3K27me3 at the nuclear periphery. **(A,B)** Wildtype myogenic progenitors or emerin null myogenic progenitors expressing WT emerin, S54F emerin, or Q133H emerin were fixed and incubated with antibodies against emerin and H3K9me2 **(A)** or H3K27me3 **(B)**. Representative images of DAPI, emerin, and H3K9me2 or H3K27me3 staining are shown. Images were analyzed using ImageJ software to measure enrichment at the nuclear envelope. A line was drawn across the nucleus in each field and fluorescence was quantified along the line. Representative line graphs are shown for each set of images (N > 30 for each cell line). Scale bars represent 10 µm.

G9a mediates the mono- and di-methylation of H3K9 (Poulard et al., 2021) and EZH2 mediates the trimethylation of H3K27 (Tan et al., 2014). Thus, we tested if enhanced binding of S54F and Q133H to G9a and EZH2 affected their activity at the nuclear envelope. Confocal immunofluorescence microscopy was used to localize emerin, EZH2, G9a, H3K9me2, and H3K27me3 in wildtype, emerin-null, S54F, and Q133H myogenic progenitors (Figure 3; Supplementary Figure S1–S12; *n* > 30 for each). A line was drawn through each nucleus and relative fluorescence along this line was plotted to monitor spatial distribution of each protein and its associated modification. H3K9me2 (Figure 3A; Supplementary Figure S3) and H3K27me3 (Figure 3B; Supplementary Figure S8) images in wildtype progenitors show greater peripheral localization of each modification when compared to cells lacking emerin. Emerin-null progenitors had less H3K9me2 (Figure 3A; Supplementary Figure S4) and H3K27me3 (Figure 3B; Supplementary Figure S9), which was rescued by expression of wildtype emerin (+EMD, Figures 3A,B; Supplementary Figure S5, S10). Both S54F and Q133H, which fail to bind HDAC3, show increased localization of H3K9me2 (Figure 3A; Supplementary Figure S6,S7) and H3K27me3 (Figure 3B; Supplementary Figure S11,12) at the nuclear periphery when compared to wildtype myogenic progenitors. G9a and EZH2 were not enriched at the nuclear envelope in wildtype progenitors nor in either of the EDMD1 mutant progenitors (data not shown), suggesting the interactions between emerin and HMTs at the nuclear envelope may be transient. These results show that an increased interaction between these HDAC3-binding mutants results in an increased proportion of H3K9me2 and H3K27me3 chromatin localized at the nuclear envelope.

### Inhibitors UNC0638 and GSK126 reduce H3K9me2 and H3K27me3 levels throughout differentiation

**FIGURE 4:**
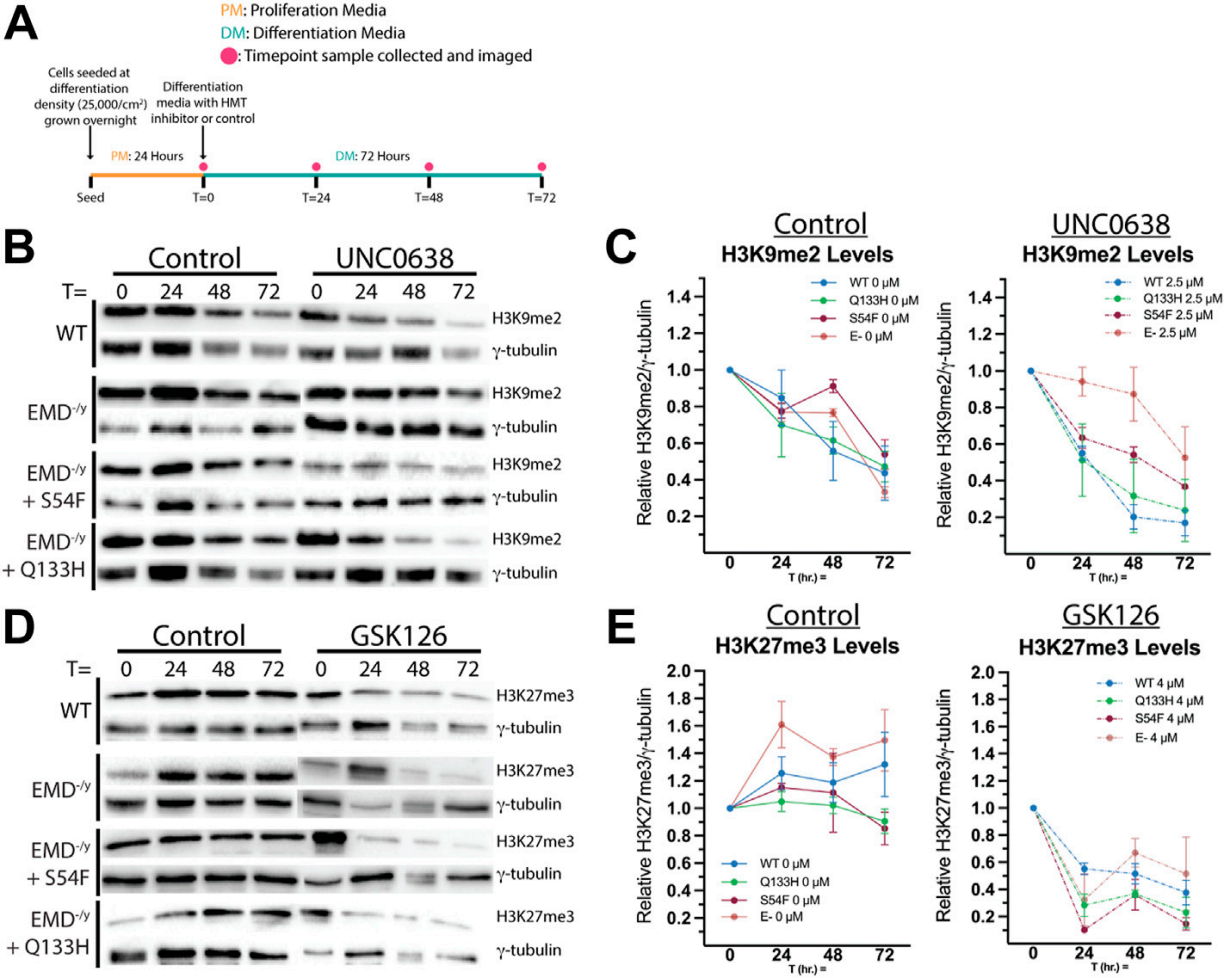
H3K9me2 and H3K27me3 are reduced by histone methyltransferase inhibitors throughout differentiation. **(A)** Schematic of the experimental approach for validating inhibitors UNC0638 and GSK126 is shown. Wildtype, emerin-null (E-), S54F, or Q133H myogenic progenitors were seeded at 25,000 cells/cm^2^ and grown overnight in proliferation media at 10% CO_2_ and 33°C. At t = 0 h, differentiation was induced by replacing the media with differentiation media and incubating at 5% CO_2_ and 37°C. Differentiation media contained 2.5 uM UNC0638, 4 uM GSK126, or ethanol alone. Images and samples were collected at 0, 24, 48, and 72 h. UNC0638 **(B)** and GSK126 **(D)** samples were separated by SDS-PAGE and visualized by western blot. Quantitation was performed *via* densitometry analysis of H3K9me2 [**(C)**; *n* = 2] and H3K27me3 [**(E)**; *n* = 2] at each time point and plotted. Bands were normalized to γ-tubulin expression. Error bars represent SEM.

We reasoned higher relative levels of H3K9me2 and H3K27me3 at the nuclear envelope in HDAC3-binding emerin mutants were due to increased binding of emerin to G9a and EZH2. Thus, we tested if this increased H3K9 and H3K27 methylation may contribute to the impaired differentiation of S54F and Q133H progenitors. We used selective inhibitors of G9a (UNC0638) and EZH2 (GSK126) to reduce H3K9me2 and H3K27me3 levels, respectively, to test this hypothesis. UNC0638 and GSK126 have reported IC_50_ values of 15 and 9.9 nM, respectively (BioVision). To validate our approach (Figure 4A), we first confirmed these inhibitors would be functional throughout differentiation (72 h) by measuring total levels of their respective histone modifications. Myogenic progenitors were plated at differentiation density, treated with UNC0638 and induced to differentiate. Cells were suspended in SDS-PAGE sample buffer at 0 h (upon introduction of differentiation media), 24 h, 48 h, and 72 h. Western-blotting was done on whole cell lysates using antibodies against H3K9me2 to confirm G9a inhibition throughout differentiation. H3K9me2 levels in wildtype, S54F and Q133H were reduced 61.3%, 31.7%, and 87.1%, after 72 h (Figures 4B,C; *n* = 2), respectively. H3K9me2 levels at 24 h showed a reduction of 35.2% in wildtype, 17.8% in S54F, and 26.7% in Q133H differentiating myogenic progenitors, demonstrating that UNC0638 remains functional throughout the differentiation assay. Treatment with UNC0638 lowered the levels of H3K9me2 at all time points, with the exception of emerin-null cells (Figure 4C). Similar experiments were done using the EZH2 inhibitor, GSK126. Inhibition of EZH2 throughout differentiation was also seen, as H3K27me3 was reduced at all time points in the presence of GSK126 for all cell lines compared to control (Figures 4D,E; *n* = 2). H3K27me3 levels in wildtype, in S54F, in Q133H, and in emerin-null cells were reduced 71.2% 68.1%, 74.7%, and 71.5% after 72 h of differentiation (Figures 4D,E), respectively. Similar reductions of H3K27me3 were seen at 24 h (64.0% in wildtype, 68.8% in S54F, 67.0% in Q133H, and 82.1% in emerin-null), showing GSK126 remains functional throughout the differentiation assay.

**FIGURE 5:**
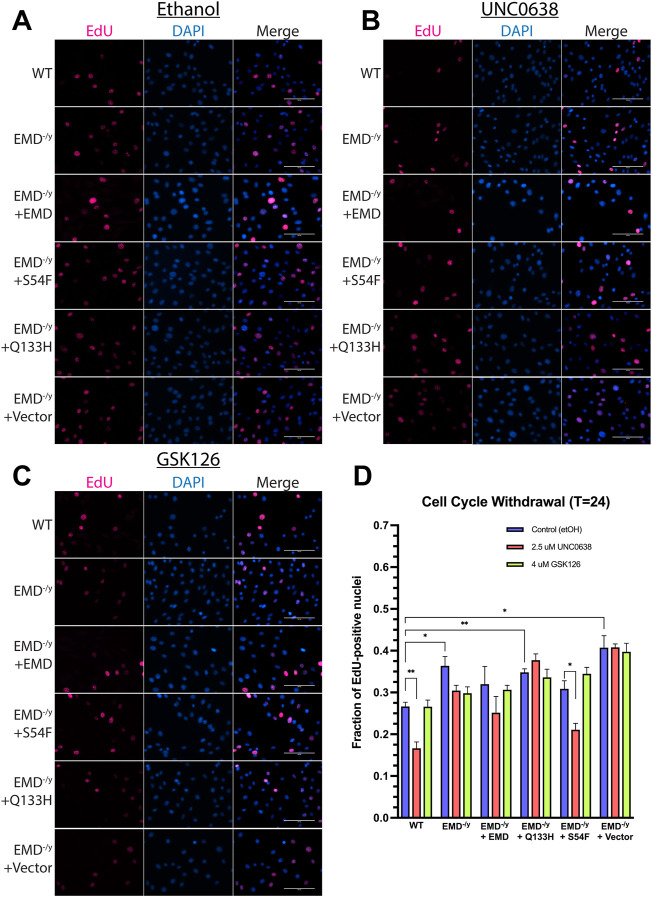
UNC0638 and GSK126 treatment fails to rescue impaired cell cycle withdrawal of emerin-null, S54F, and Q133H myogenic progenitors. The indicated myogenic progenitor cell lines were induced to differentiate in the presence of ethanol alone **(A)**, UNC0638 **(B)** or GSK126 **(C)** for 24 h. Cells were incubated with EdU for 2 h prior to fixing and imaging to monitor cell cycle withdrawal. EdU-positive cells were counted and plotted [**(D)**; *n* > 225 for each cell line]. Scale bars represent 100 μm. Error bars represent SEM. (Student’s t-test;*p < 0.05 **p < 0.01)

Now that we confirmed functionality of UNC0638 and GSK126 throughout differentiation, we wanted to test the impact of G9a or EZH2 inhibition on wildtype and EDMD1 mutant myogenic differentiation. EdU incorporation was measured 24 h after differentiation induction to assess cell cycle withdrawal. G9a inhibition increased cell cycle withdrawal during differentiation of wildtype and S54F progenitors (Figures 5B,D, *n* = 3) but failed to rescue cell cycle withdrawal during differentiation of Q133H and emerin-null progenitors (Figures 5B,D; *n* = 3). EZH2 inhibition failed to significantly alter cell cycle exit in any of the progenitor lines tested compared (Figures 5C,D; *n* = 3).

**FIGURE 6:**
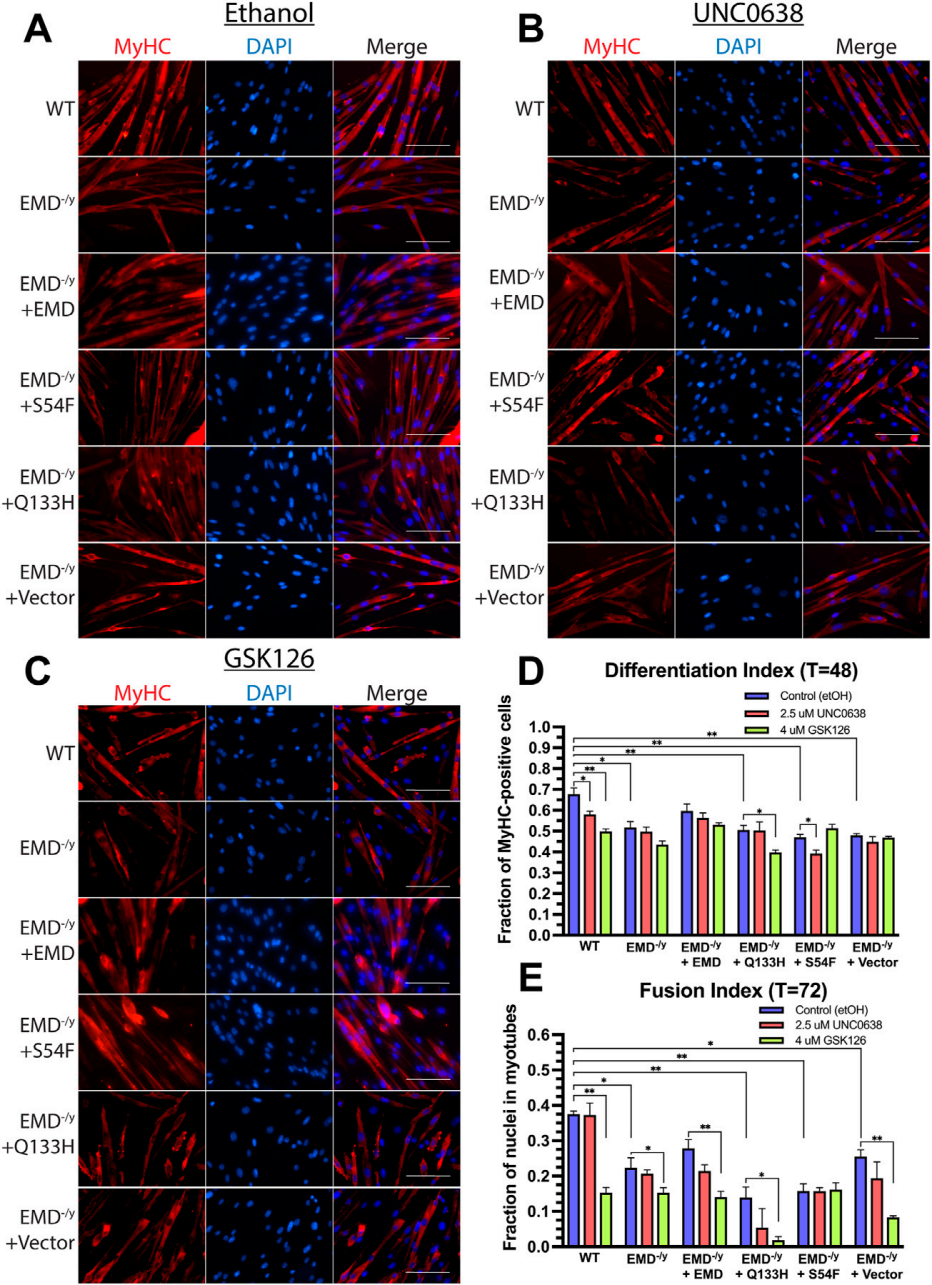
Inhibition of G9a and EZH2 fails to rescue emerin-null, S54F, and Q133H myogenic progenitor differentiation, but exacerbates their differentiation defects. The indicated myogenic progenitor cell lines were induced to differentiate in the presence of ethanol alone **(A)**, UNC0638 **(B)**, or GSK126 **(C)** for 48 or 72 h. Cells were fixed, incubated with antibodies against MyHC, and stained with DAPI. The differentiation index **(D)** is the number of nuclei in MyHC^+^ multinucleated myotubes plus the number of nuclei in MyHC mono- or di-nuclear cells/total nuclei after 48 h. The fusion index **(E)** is the number of nuclei in MyHC^+^ multi-nucleated myotubes (≥3 nuclei/total nuclei) after 72 h. Scale bars represent 100 µm. Error bars represent SEM. (Student’s t-test; *p < 0.05 **p < 0.01).

We next measured the differentiation index (number of nuclei in MyHC^+^ multinucleated myotubes + number of nuclei in MyHC^+^ mono- or di-nuclear cells/total nuclei) and the fusion index (number of nuclei in MyHC^+^ multi-nucleated myotubes (≥3 nuclei/total nuclei)) to assess myoblast commitment and fusion to form myotubes, respectively, in the presence of UNC0638 and GSK126. To monitor myoblast commitment and fusion, expression of MyHC was detected by immunofluorescence microscopy 48 h after differentiation induction, while the fusion index is determined at 72 h post-induction. G9a inhibition significantly decreased the differentiation index in wildtype emerin and S54F mutant cells (Figures 6B,D; *n* = 3), while no significant difference was detected in emerin-null and Q133H mutant cells (Figures 6B,D). Inhibition of EZH2 reduced the differentiation index of wildtype emerin and Q133H mutant cells, while no significant change was detected in emerin-null and S54F mutant cells (Figures 6C,D; *n* = 3). The fusion index was significantly decreased in emerin-null and Q133H mutant cells upon treatment with UNC0638 (Figures 6B,E; *n* = 3), while no effect was seen in emerin-null and S54F mutant cells. Treatment with GSK126 decreased myotube formation in wildtype emerin, emerin-null and Q133H mutant cells (Figures 6C,E; *n* = 3), while no significant difference was seen in S54F mutant cells.

## Discussion

The myogenic differentiation program is a coordinated process dependent upon epigenetic mechanisms to regulate proper gene expression, including modulating histone modifications (Bharathy et al., 2013), RNA-mediated silencing (Bianchi et al., 2017), and DNA methylation (Yang et al., 2021) at specific gene loci. In differentiating adult stem cells, coordinated repression and activation of developmentally regulated loci occurs by association of chromatin with the nuclear lamina at the nuclear envelope (Wong et al., 2014; van Steensel and Belmont, 2017). These Lamina Associated Domains (LADs) consist of both developmentally regulated genes (facultative LADs; fLADs) and constitutively inactive domains (constitutive LADs; cLADS) (Meuleman et al., 2013; Zheng et al., 2015). fLADs consist of developmental genes that alter their association with the nuclear lamina during differentiation and are enriched for the histone modifications H3K9me2 and H3K27me3 (Luperchio et al., 2014; Kind et al., 2015). fLAD reorganization is seen during tissue-specific differentiation resulting in the coordinated temporal expression of key differentiation genes in neuronal differentiation (Peric-Hupkes et al., 2010), myogenic differentiation (Demmerle et al., 2013), and cardiac progenitor differentiation (Poleshko et al., 2017).

Increasing evidence demonstrates the importance of epigenetic modifications on cell fate, but the mechanism by which repressive chromatin is dynamically organized at the nuclear periphery remains unclear. Previous work in our lab and by others have shown emerin is an important regulator of chromatin organization through its interaction with, and activation of, HDAC3 (Demmerle et al., 2012, 2013; Collins et al., 2017). Emerin and HDAC3 are required for localizing LADs to the nuclear envelope to repress transcription (Demmerle et al., 2012, 2013). Targeting H4K5 acetylation or deacetylation by pharmacological activators or inhibitors to decrease total H4K5ac levels in emerin-null myogenic progenitors rescued their differentiation defects (Collins et al., 2017; Bossone et al., 2020). This suggests histone modifications are important in regulating myogenic fLAD organization. Nuclear envelope-localized HDAC3 is also required for cardiomyocyte differentiation (Poleshko et al., 2017), suggesting this mechanism may be conserved in adult stem cell differentiation. Methylation of H3K9 was also shown to be important for LAD localization (Towbin et al., 2012). H3K9me2 and H3K27me3 are the products of G9a (Shinkai and Tachibana, 2011) and EZH2 (Tan et al., 2014), respectively, and inhibition of these HMTs disrupts LAD formation (Towbin et al., 2012; Harr et al., 2015). The mechanism of HMT recruitment to LADs remains unclear, but HDAC3 was shown to recruit EZH2 to repressive loci (Wang et al., 2011; Zhang et al., 2012; Emmett and Lazar, 2019) and emerin was shown to recruit EZH2 and H3K27me3 to the nuclear periphery in C2C12 myoblasts and 293T cells (Ma et al., 2019).

In this study, we show emerin binds G9a and EZH2 in wildtype progenitors and that this interaction is enhanced in the HDAC3-binding mutant progenitors S54F and Q133H. We next showed H3K9me2 and H3K27me3 are enriched at the nuclear periphery in these same mutants. We then chose to target catalytic activity of both G9a and EZH2 with small molecule inhibitors. We demonstrate that inhibition of either histone methyltransferase failed to rescue commitment or myotube formation to wildtype levels in emerin-null cells expressing Q133H or S54F. Enrichment of H3K9me2 and H3K27me3 at the nuclear periphery upon increased emerin-HMT binding in the emerin HDAC3-binding mutants suggests emerin is important for deposition of these marks or for recruitment of chromatin containing these marks to the nuclear envelope.

**FIGURE 7:**
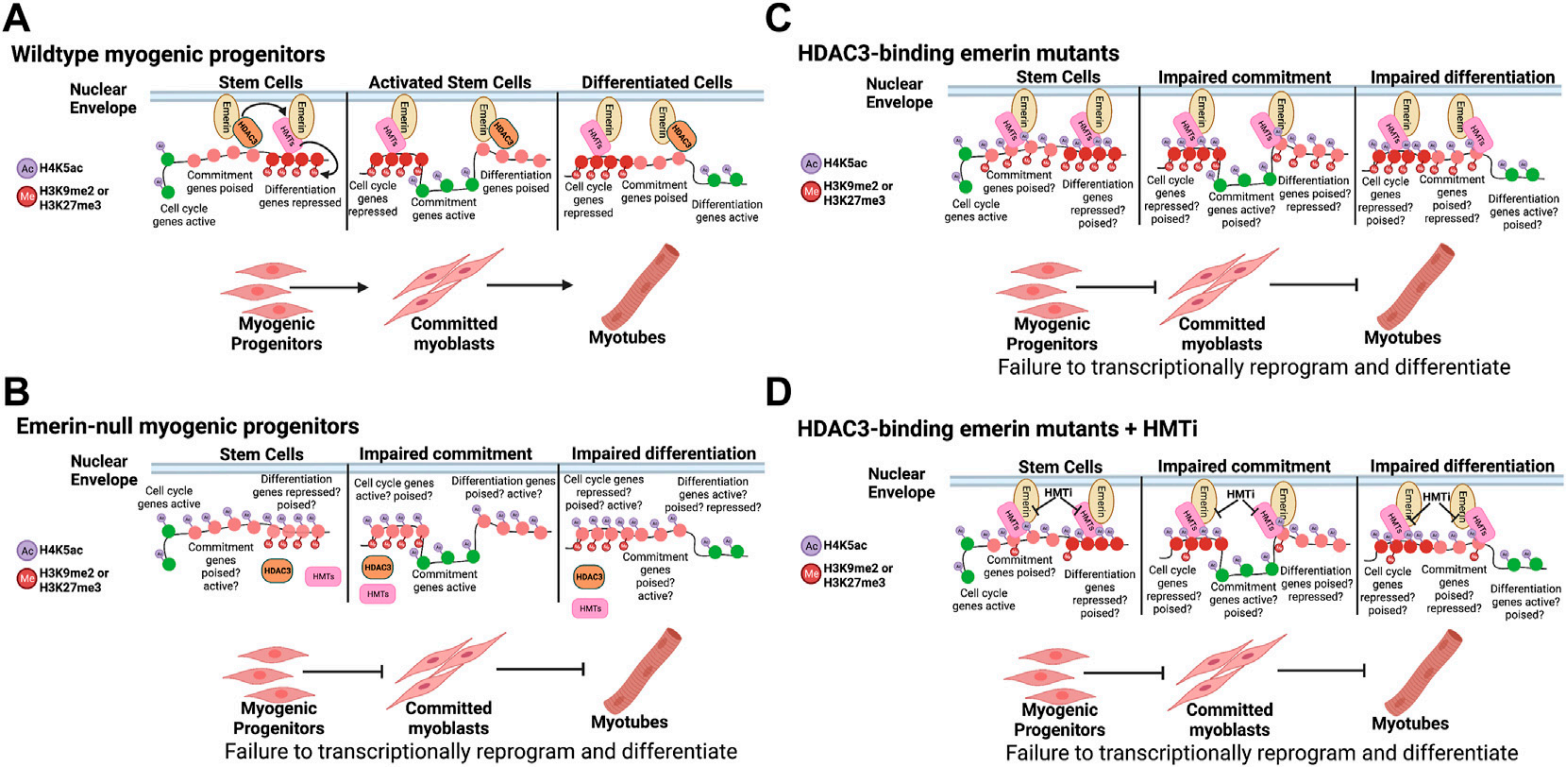
Model. **(A)** Wildtype myogenic progenitors dynamically reorganize their genome to coordinate the temporal expression of the differentiation program. **(B)** Cells lacking emerin fail to coordinate the temporal expression of key myogenic loci due to failure to dynamically reorganize the genome. **(C)** HDAC3-binding emerin mutants are predicted to exhibit dually acetylated and methylated chromatin states on key myogenic loci, leading to failure to coordinate their temporal expression. **(D)** Failure of HMT inhibition to rescue differentiation of HDAC3-binding emerin mutants is predicted to be due to the hyperacetylation of H4K5 on key myogenic loci independent of their methylation status. This is consistent with the role of HDAC3 in fLAD organization during myogenic and cardiomyogenic differentiation (Demmerle et al., 2012, 2013; Poleshko et al., 2017). This figure was created using BioRender software.

The emerin-HDAC3 interaction is important for the coordinated temporal relocalization of fLADs during myogenic differentiation (Demmerle et al., 2013). However, altering HMT activity fails to rescue differentiation, suggesting these changes are not important for myogenic differentiation. This supports a model whereby emerin helps maintain stably repressed H3K9me2/3 and H3K27me3 cLADs at the nuclear envelope through its interactions with HMTs. In this model (Figure 7) it is the interaction of emerin, as well as other unidentified nuclear envelope proteins, with HMTs that help establish cLADs, while the emerin-HDAC3 interaction modulates the dynamic localization of key myogenic loci (fLADs), including *MyoD*, *Myf5*, *Pax3/7*. Here, wildtype myogenic progenitors nuclear envelope-associated HMTs help organize cLADs (Figure 7A; H3K9me2/H3K27me3) at the nuclear lamina. In emerin mutants that disrupt HDAC3 binding, HDAC3 fails to localize to the periphery resulting in greater EZH2/G9a at the nuclear envelope, and by extension, increased H3K9me2 and H3K27me3 at the nuclear envelope (Figure 7C). In this model, increased H3K9me2 and H3K27me3 would be coincident with increases in H4K5ac at the nuclear envelope, since HDAC3 is not present at the periphery. When HMT activity is inhibited, this blocks methylation of H3K9 or H3K27, which we predict preferentially affects cLAD organization (Figure 7D). The lack of HDAC3 at the nuclear envelope would still result in hyperacetylation of LADs, which we predict are specifically important for fLAD localization (Figure 7D). This is supported by the failure of myogenic fLADs (e.g., *Myf5*) to localize correctly in emerin-null or emerin S54F mutant progenitors and its failure to rescue differentiation (Demmerle et al., 2013; Iyer and Holaska, 2020). In this model the increased, aberrant H4K5 acetylation of the myogenic fLADs results in their misexpression regardless of HMT activity at the nuclear envelope (Figures 7C,D). However, we do not know whether these fLADs are methylated at H3K9 or H3K27 in these myogenic progenitors and thus are similar to ‘poised genes’ or if they lack H3K9 or H3K27 methylation and resemble transcriptionally active genes. Future studies will identify post-translational modifications at these myogenic fLADs to examine these possibilities.

It is possible that HMTs could be important for fLAD organization and HDAC3 could be important for its reorganization, as our studies and our proposed model fail to differentiate between these possibilities. For example, emerin-null and mutant progenitors have reduced global H3K27me3 and H3K9me2/3—a potential contributor to their impaired differentiation. Future studies will be necessary to investigate the effect of increasing global H3K9 and H3K27 methylation using specific demethylase inhibitors. More experiments are also necessary to examine how specific myogenic loci are dynamically localized and how this correlates with their specific chromatin post-translational modification states in emerin mutant myogenic progenitors.

Although these and our previous studies (Demmerle et al., 2013; Bossone et al., 2020; Iyer and Holaska, 2020) support this model (Figure 7), there are other models that need to be considered. One such model would be that HDAC3 catalytic activity is dispensable for fLAD formation or localization, as shown in cardiomyocytes (Poleshko et al., 2017), suggestive that the role of HDAC3 is to tether repressed chromatin. Similarly, HMTs may function as a tether for repressed chromatin. In this regard, HDAC3-binding emerin mutants are able to bind more HMTs and therefore localize more H3K9me2/3 or H3K27me3 to the nuclear envelope. If HMT binding to emerin at the nuclear periphery renders them catalytically inactive, we would expect decreased total amounts of H3K9me2/3 and H3K27me3 in the absence of emerin or HDAC3 which is consistent with pervious data (Knutson et al., 2008; Bhaskara et al., 2010; Demmerle et al., 2013; Bossone et al., 2020). A second model would be that methylation of H3K9 and H3K27 may require deacetylation of H4K5 to recruit HMTs independent of any HDAC3-HMT interaction. It is also possible that two distinct complexes form containing emerin-HDAC3-fLADs and emerin-HMT-cLADs. A third model would be that HDAC3 stimulates nuclear envelope recruitment of H3K9me2 by deacetylating H3K9ac. Although HDAC3 has not been shown to directly deacetylate H3K9, we and others have shown that loss of HDAC3 or emerin results in increased H3K9ac and decreased H3K9me2/3 (Knutson et al., 2008; Bhaskara et al., 2010; Demmerle et al., 2013). Future studies are needed to determine which model(s) is correct.

It is unclear from these studies as to whether emerin binds directly to EZH2 or G9a. Emerin may bind EZH2 and G9a through binding to their respective complex components. EZH2 and G9a may also interact with emerin through other known binding proteins. One candidate protein is Msx1. Emerin was shown to bind Msx1 and bridge an interaction between EZH2 and emerin (Ma et al., 2019). G9a was also shown to interact with Msx1 (Wang and Abate-Shen, 2012a), so it is possible Msx1 mediates the binding of emerin to HMTs.

The functional interaction of emerin with EZH2 and Msx1 is important for myogenic differentiation. Emerin knockdown in proliferating C2C12 myoblasts was shown to displace EZH2 and H3K27me3 from the nuclear periphery (Ma et al., 2019), suggesting emerin binding to EZH2 organizes repressive chromatin at the periphery (Wang and Abate-Shen, 2012b). Further, the catalytic activity of EZH2 was essential for myogenic differentiation (Caretti et al., 2004). These results are consistent with previous experiments showing myoblasts treated with EZH2 shRNA exhibit impaired differentiation and delayed the expression of myogenic genes (Adhikari and Davie, 2018). One pathway in which EZH2 is important for myogenic differentiation is the Wnt pathway (Markiewicz et al., 2006; Iyer et al., 2017; Iyer and Holaska, 2020). The Wnt pathway is activated after muscle injury to regulate myogenic differentiation (Brack et al., 2008) and is regulated by EZH2 and the PRC2 complex (Adhikari et al., 2019) by inhibition of Wnt antagonists. Interestingly, the Wnt pathway is dysfunctional in emerin-null and emerin mutant myogenic progenitors (Iyer et al., 2017; Iyer and Holaska, 2020).

Although the relative levels of H3K9me2 and H3K27me3 at the nuclear envelope are increased in HDAC3-binding emerin mutants, we do not observe more EZH2 or G9a at the nuclear envelope. This could be due to epitope masking of EZH2 or G9a upon binding to emerin at the nuclear envelope. Alternatively, it is possible that in live cells the interactions between EZH2 or G9a and emerin are more transient, as might be expected for an enzyme. This transient interaction would likely be sufficient to add the H3K9me2/3 or H3K27me3 modification. In this scenario, the conditions of the immunoprecipitation may increase the stability of the interactions between emerin and EZH2 that occurs in cells. Lastly, the interaction between emerin, Msx1, and G9a or EZH2 may mask the epitopes of G9a or EZH2 only when bound to Msx1 in cells. In this scenario more binding of G9a or EZH2 to emerin *via* Msx1 would be seen *in vitro*, but this increase would be masked in cells. Expression of exogenous G9a or EZH2 will be used in future experiments to test these possibilities.

In summary, it is clear the coordinated temporal reorganization of the genome occurs progressively, as some myogenic differentiation genes become active while cell cycle regulation and mitotic genes are silenced. Thus, this process occurs through multiple steps, all of which are crucial for proper differentiation into myotubes. It is possible that the failure to rescue myotube formation we observed in G9a and EZH2 inhibition experiments is a consequence of enriched H4K5ac from loss of HDAC3 interaction in S54F, Q133H, or emerin-null progenitors. One potential explanation would be that deacetylation of H4K5 is a critical step in the myogenic differentiation program. Further, the binding of HDAC3 to these regions would help limit the amount of more stable chromatin repression by limiting the amount of EZH2 or G9a at the nuclear envelope, thereby allowing precise control of the regions to be methylated with H3K9me2 or H3K27me3. In this way, emerin may also be important for maintaining homeostasis of H3K9me2 and H3K27me3 localization at the nuclear periphery, as well as its dynamic reorganization during differentiation. We posit that the interaction of S54F or Q133H emerin with EZH2 or G9a-containing complexes lack proper regulation due to their inability to bind HDAC3, resulting in defective chromatin organization at the nuclear periphery. Cells lacking emerin also exhibit a decrease in repressive marks, likely due to this inability to properly regulate repressive chromatin organization and are thus unable to appropriately repress cell cycle genes. Previous data from our lab and the experiments in this study strongly suggest emerin plays multiple roles in regulating dynamic genomic organization at the nuclear periphery.

## Data Availability

The raw data supporting the conclusion of this article will be made available by the authors, without undue reservation.
