# Supplementary figures and images for: Emerin interacts with histone methyltransferases to regulate repressive chromatin at the nuclear periphery
Source: Front Cell Dev Biol. 2022 Oct 6;10:1007120. doi: 10.3389/fcell.2022.1007120 (PMC9583931; doi:10.3389/fcell.2022.1007120)

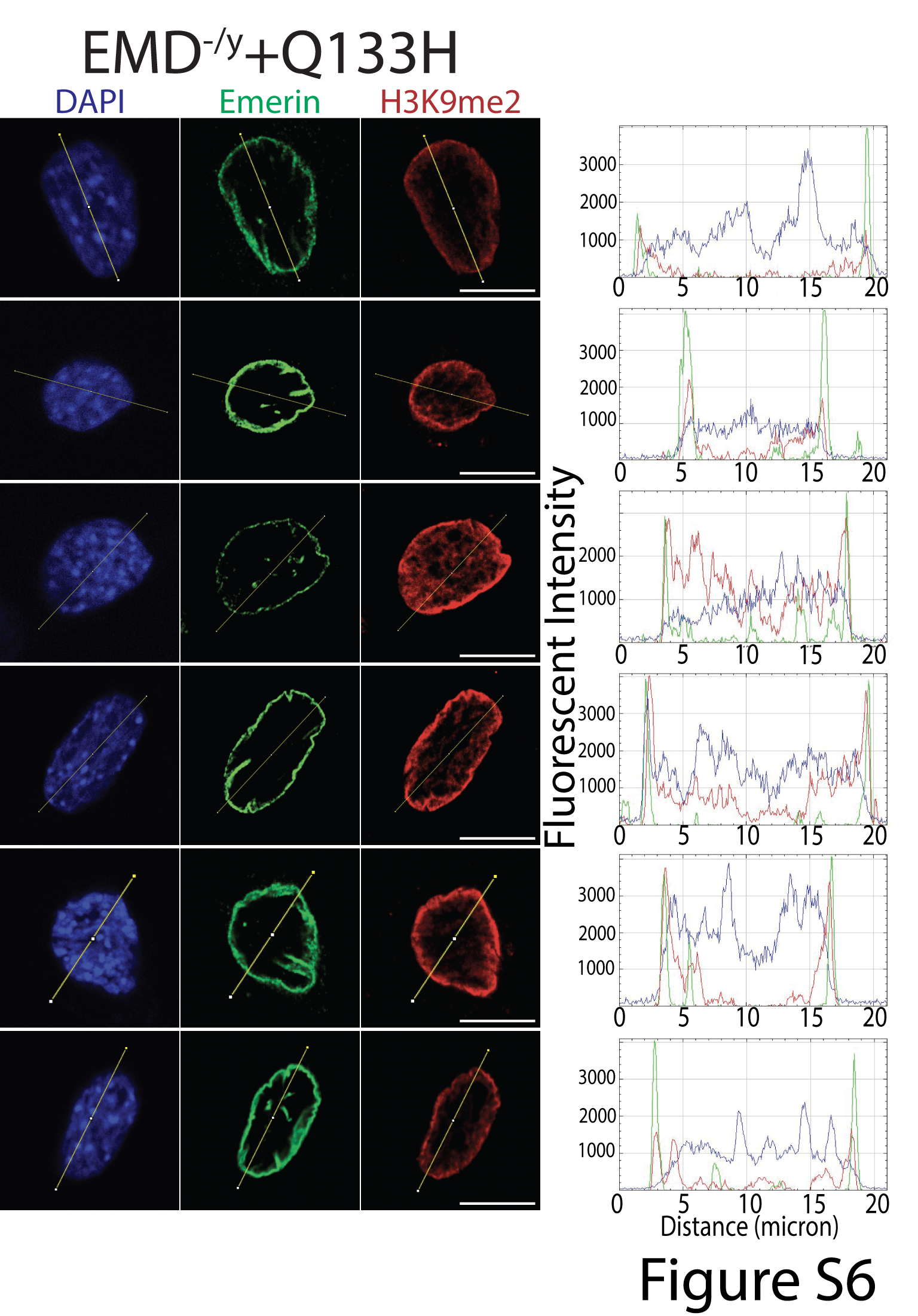

Supplement: Supplementary file 1 [file Image6.TIF]

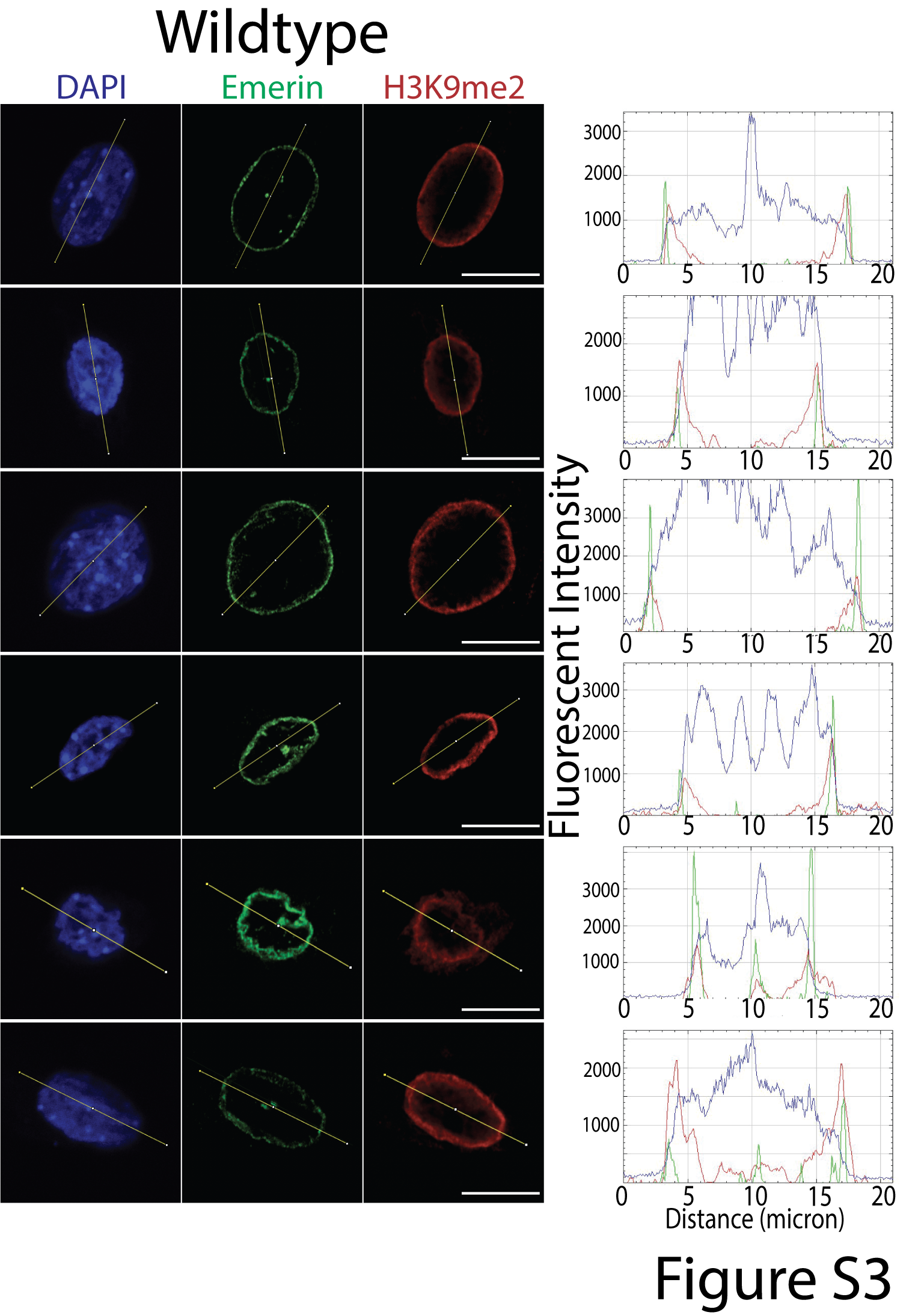

Supplement: Supplementary file 2 [file Image3.TIF]

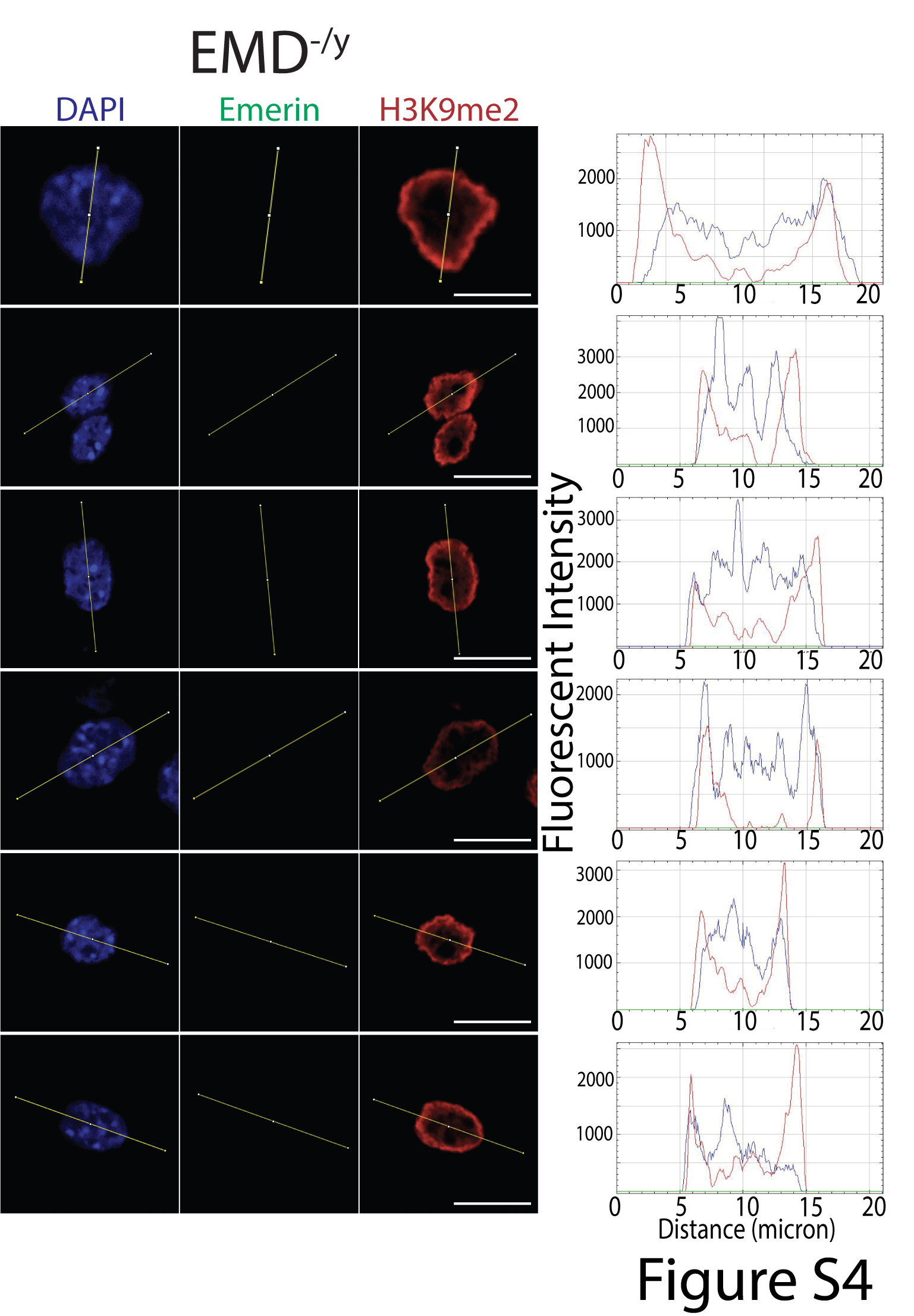

Supplement: Supplementary file 3 [file Image4.TIF]

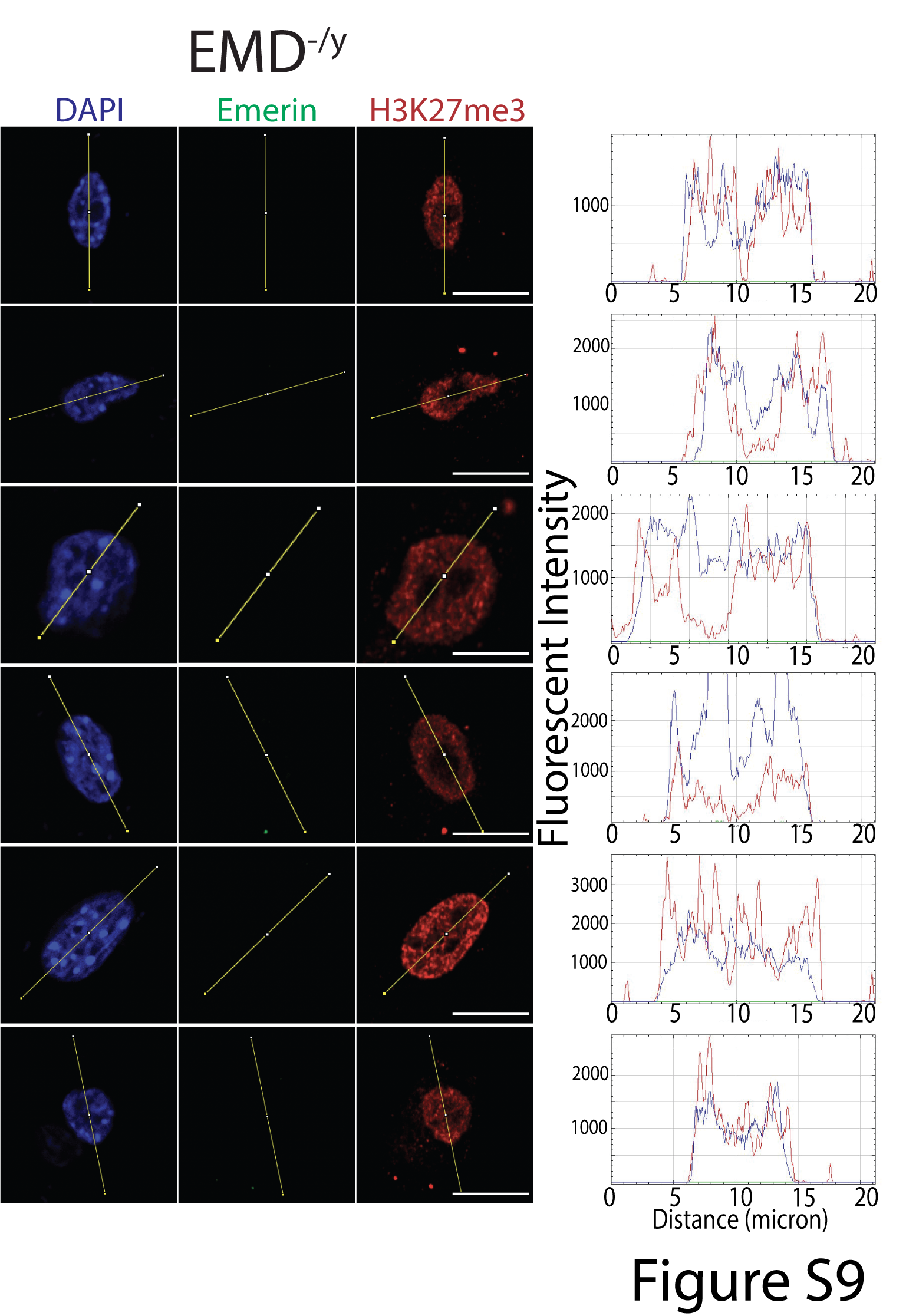

Supplement: Supplementary file 4 [file Image9.TIF]

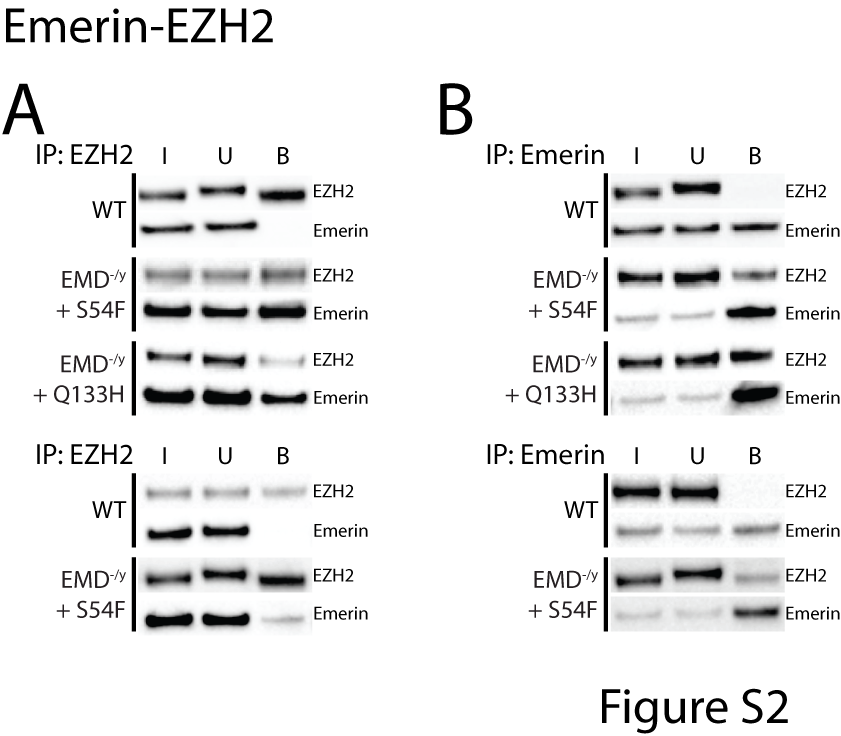

Supplement: Supplementary file 5 [file Image2.TIF]

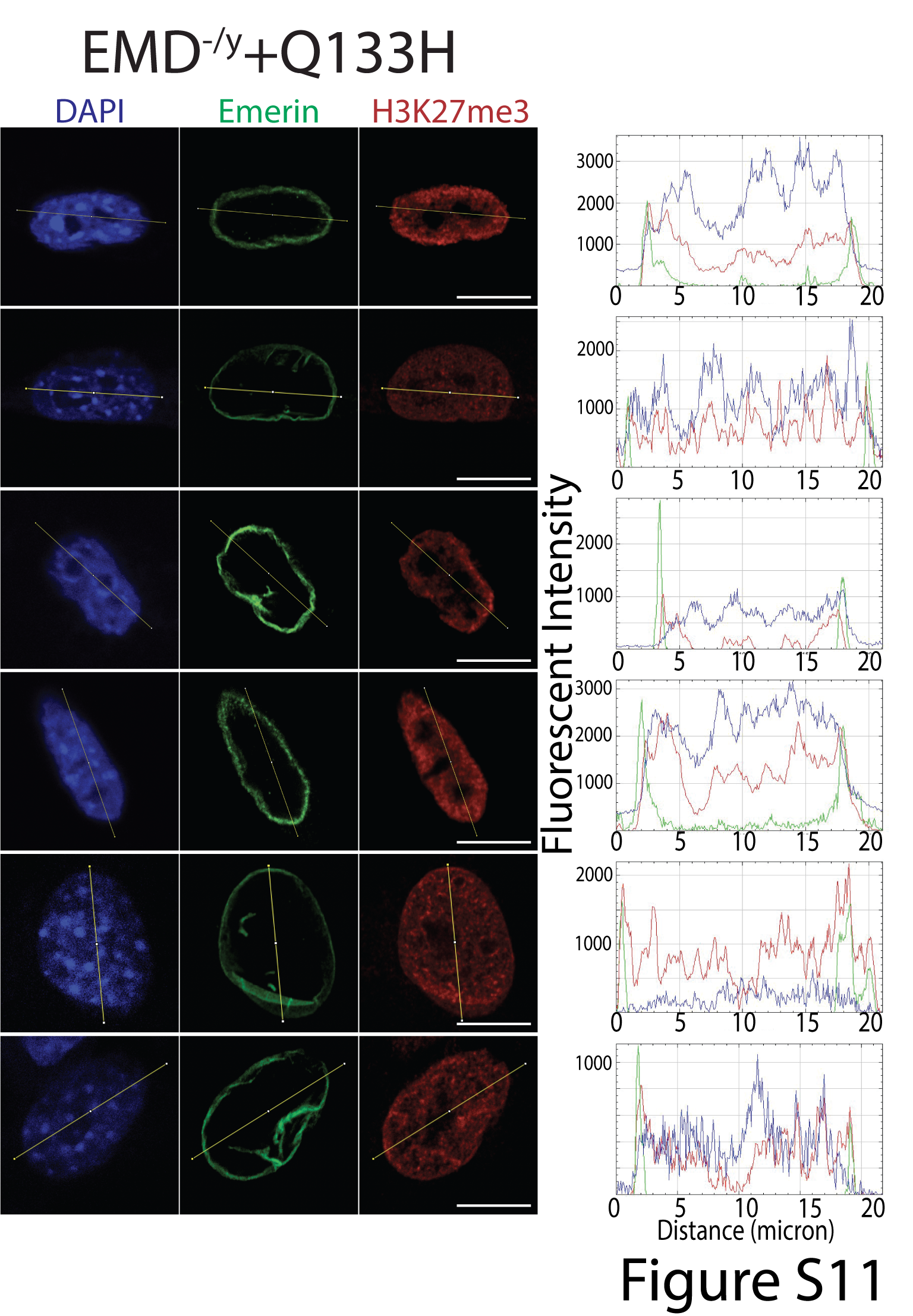

Supplement: Supplementary file 6 [file Image11.TIF]

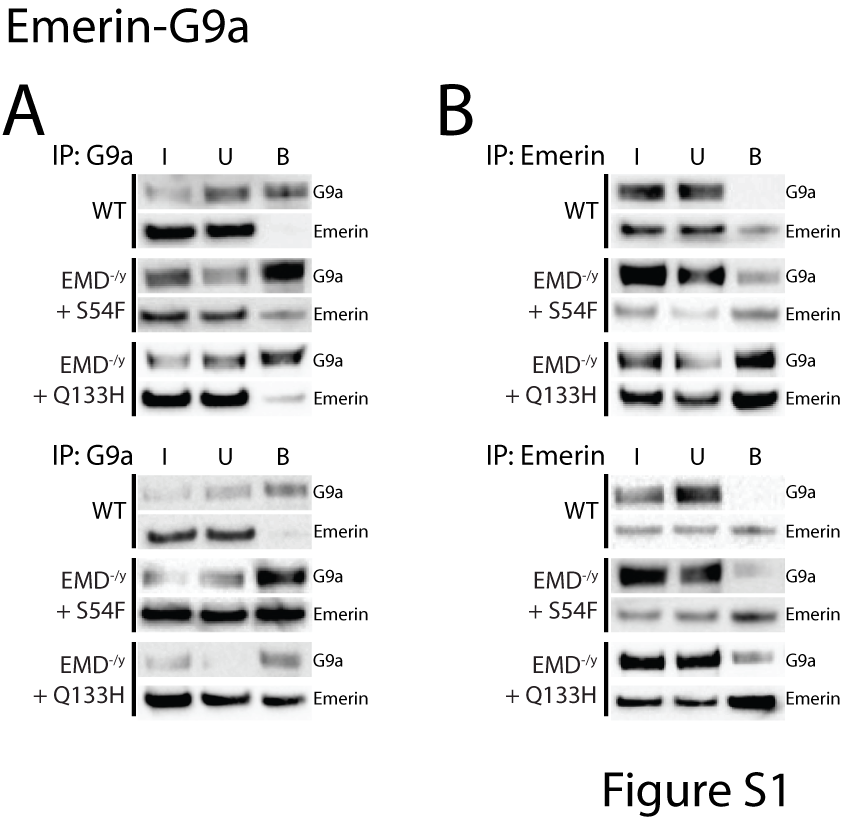

Supplement: Supplementary file 7 [file Image1.TIF]

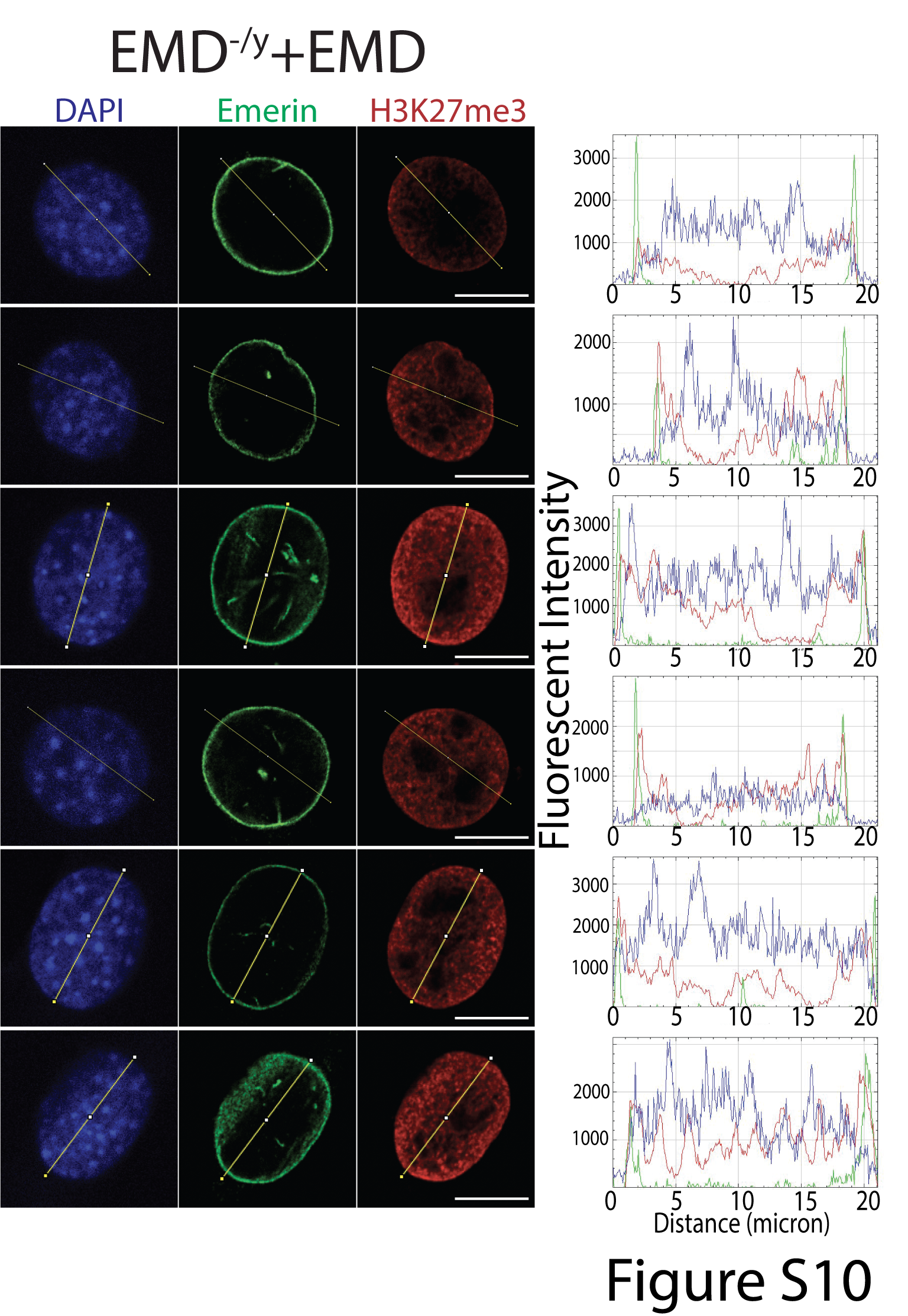

Supplement: Supplementary file 8 [file Image10.TIF]

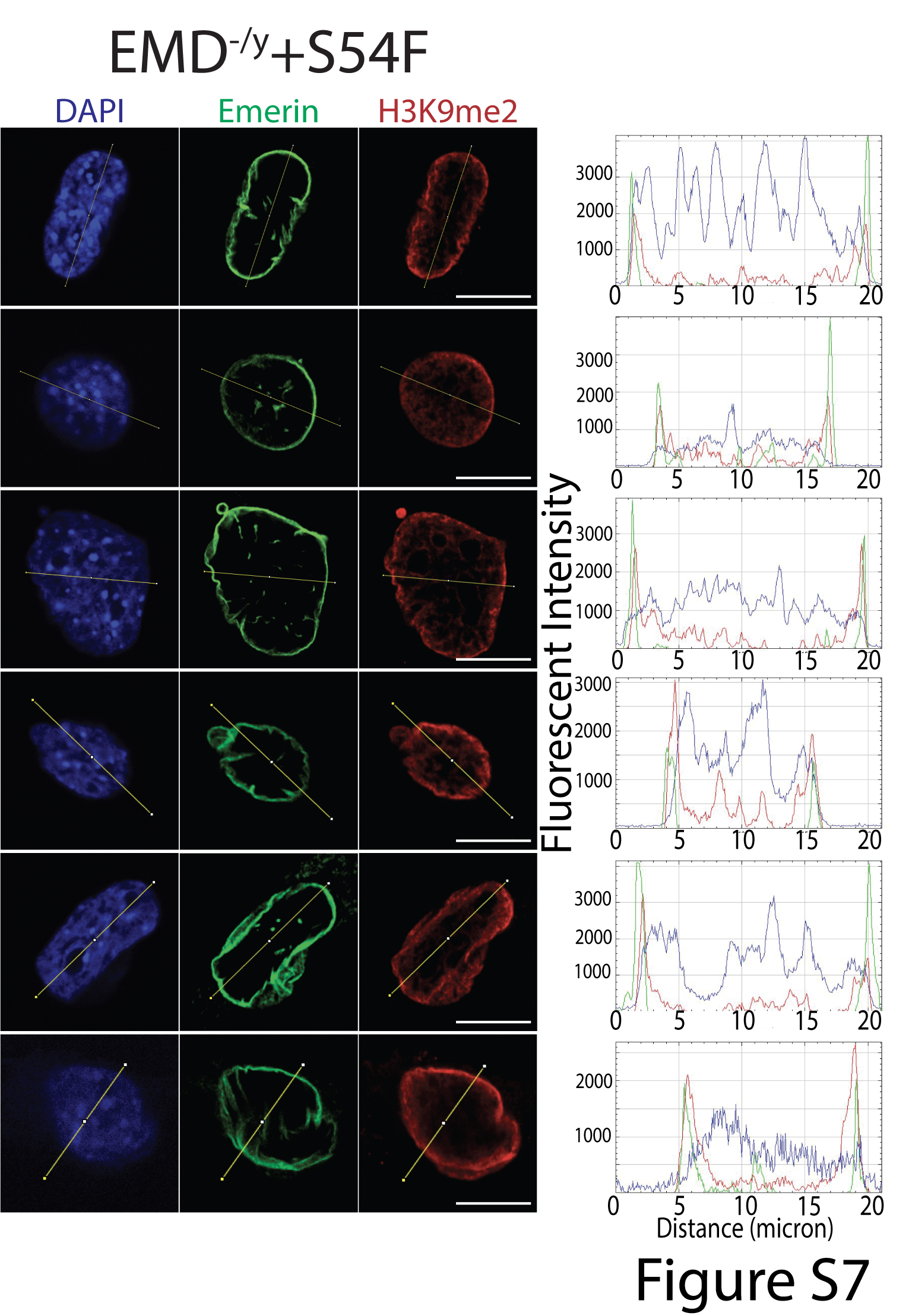

Supplement: Supplementary file 9 [file Image7.TIF]

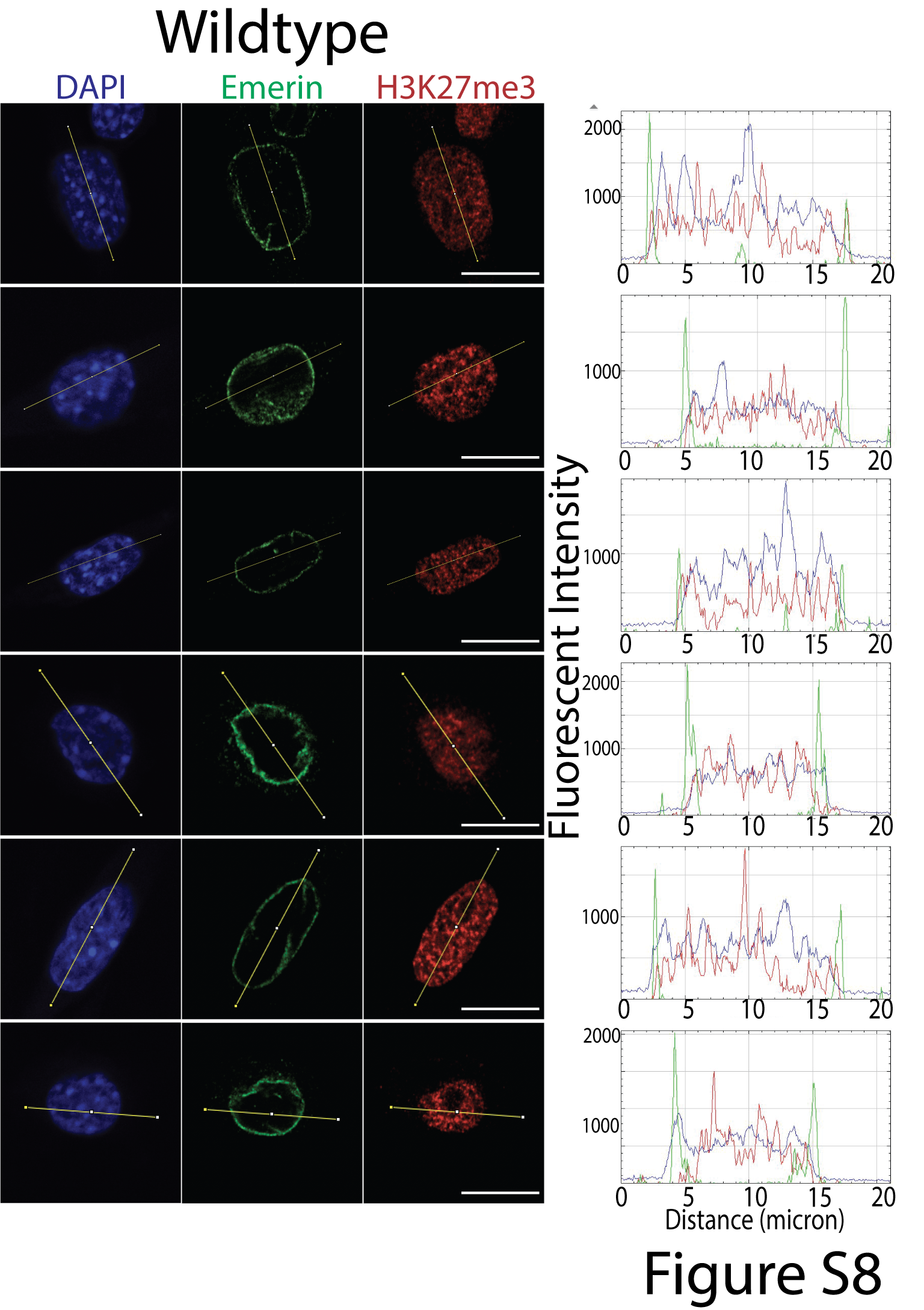

Supplement: Supplementary file 10 [file Image8.TIF]

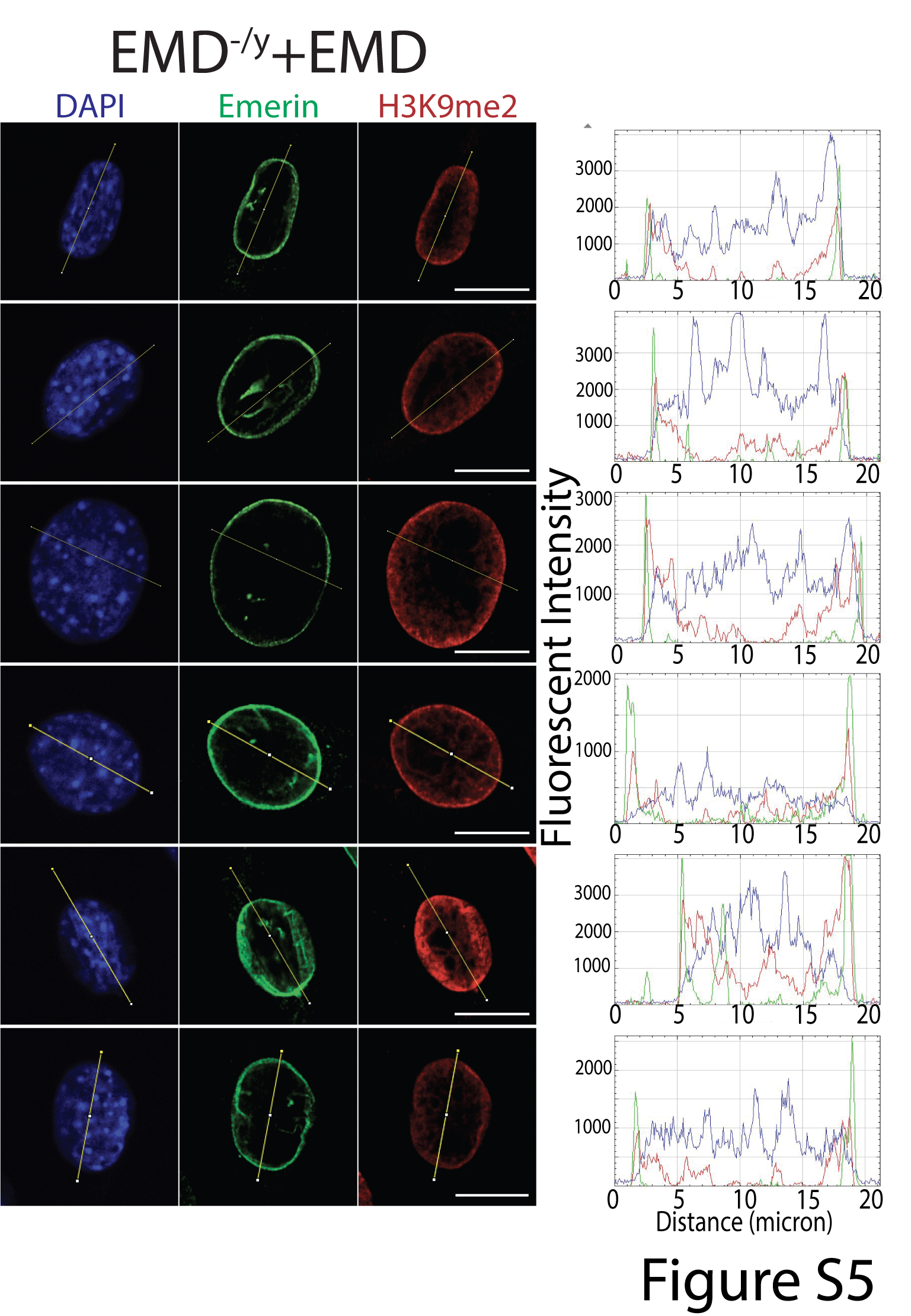

Supplement: Supplementary file 11 [file Image5.TIF]

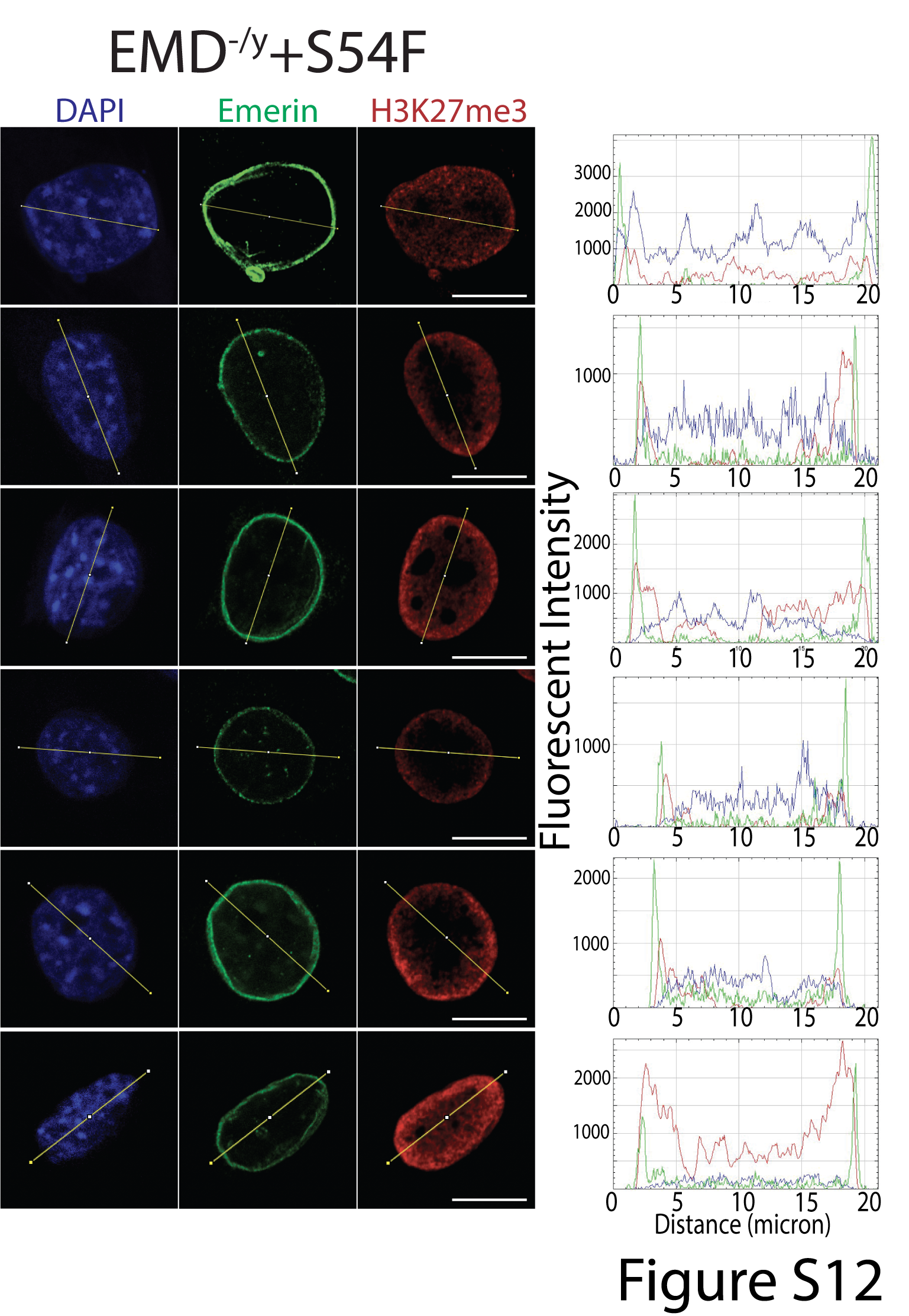

Supplement: Supplementary file 12 [file Image12.TIF]
